# Bat and pig IFN-induced transmembrane protein 3 restrict cell entry by influenza virus and lyssaviruses

**DOI:** 10.1099/vir.0.000058

**Published:** 2015-05

**Authors:** Camilla T. O. Benfield, Sarah E. Smith, Edward Wright, Rachael S. Wash, Francesca Ferrara, Nigel J. Temperton, Paul Kellam

**Affiliations:** ^1^​ Department of Pathology and Pathogen Biology, The Royal Veterinary College, Hatfield, UK; ^2^​ Wellcome Trust Sanger Institute, Wellcome Trust Genome Campus, Hinxton, Cambridge, UK; ^3^​ Viral Pseudotype Unit (Fitzrovia), Faculty of Science and Technology, University of Westminster, London, UK; ^4^​ Viral Pseudotype Unit (Medway), School of Pharmacy, University of Kent, Chatham Maritime, Kent, UK; ^5^​ MRC/UCL Centre for Medical Molecular Virology, Division of Infection and Immunity, University College London, London, UK

## Abstract

IFN-induced transmembrane protein 3 (IFITM3) is a restriction factor that blocks cytosolic entry of numerous viruses that utilize acidic endosomal entry pathways. In humans and mice, IFITM3 limits influenza-induced morbidity and mortality. Although many IFITM3-sensitive viruses are zoonotic, whether IFITMs function as antiviral restriction factors in mammalian species other than humans and mice is unknown. Here, IFITM3 orthologues in the microbat (*Myotis myotis*) and pig (*Sus scrofa domesticus*) were identified using rapid amplification of cDNA ends. Amino acid residues known to be important for IFITM3 function were conserved in the pig and microbat orthologues. Ectopically expressed pig and microbat IFITM3 co-localized with transferrin (early endosomes) and CD63 (late endosomes/multivesicular bodies). Pig and microbat IFITM3 restricted cell entry mediated by multiple influenza haemagglutinin subtypes and lyssavirus glycoproteins. Expression of pig or microbat IFITM3 in A549 cells reduced influenza virus yields and nucleoprotein expression. Conversely, small interfering RNA knockdown of IFITM3 in pig NPTr cells and primary microbat cells enhanced virus replication, demonstrating that these genes are functional in their species of origin at endogenous levels. In summary, we showed that IFITMs function as potent broad-spectrum antiviral effectors in two mammals – pigs and bats – identified as major reservoirs for emerging viruses.

## Introduction

Restriction factors are germline-encoded proteins that function in a cell-autonomous manner to suppress virus replication. The IFN-induced transmembrane (IFITM) proteins are a family of small IFN-stimulated proteins that affect diverse cellular processes (reviewed by Siegrist *et al.*, 2011) and were recently identified as antiviral restriction factors that inhibit the cell entry of multiple pathogenic viruses (Brass *et al.*, 2009).

To date, IFITMs have been reported to restrict the enveloped viruses influenza A, West Nile virus and dengue virus (family *Flaviviridae*), severe acute respiratory syndrome coronavirus, Ebola virus and Marburg virus (family *Filoviridae*), vesicular stomatitis virus (VSV) and lyssaviruses (family *Rhabdoviridae*), human immunodeficiency virus type 1 and several species of the family *Bunyaviridae*, as well as a non-enveloped orthoreovirus (family *Reoviridae*) (Anafu *et al.*, 2013; Brass *et al.*, 2009; Huang *et al.*, 2011; Jia *et al.*, 2012; Lu *et al.*, 2011; Mudhasani *et al.*, 2013; Smith *et al.*, 2013; Weidner *et al.*, 2010). The common feature of IFITM-sensitive viruses appears to be their dependence on acidic endosomal entry pathways, either for proteolytic cleavage, or for pH- or protease-dependent activation of viral entry proteins into their fusogenic form. Accordingly, pseudotyped retroviruses expressing heterologous surface envelope proteins recapitulate the IFITM sensitivity of the authentic virus from which the envelope protein derives, and have been widely used to study IFITM biology (Brass *et al.*, 2009; Feeley *et al.*, 2011; Huang *et al.*, 2011). IFITMs localize to membranes of late endosomes and lysosomes and prevent the release of viral particles from these compartments into the cytosol (Feeley *et al.*, 2011).

The human *IFITM* gene family comprises *IFITM1*, -*2*, -*3* and -*5*, all of which possess antiviral activity and cluster together on chromosome 11, as well as *IFITM10* whose function remains unknown. Mice possess orthologues of all the human *IFITM* genes and two additional genes, *Ifitm6* and -*7*. IFITM1, -2 and -3 are expressed in a wide range of tissues (Bailey *et al.*, 2012; Everitt *et al.*, 2013; Siegrist *et al.*, 2011) whereas IFITM5 expression is limited to osteoblasts (Moffatt *et al.*, 2008). Of all the IFITMs, IFITM3 is the most potent anti-influenza effector *in vitro* (Huang *et al.*, 2011). Ifitm3 has a critical role in limiting influenza-induced morbidity and mortality in mice (Everitt *et al.*, 2012). As the phenotype of influenza-infected *Ifitm3*^−/−^ mice is indistinguishable from that of mice deleted for the entire locus (comprising *Ifitm1*, -*2*, -*3*, -*5* and -*6*), Ifitm3 apparently dominates influenza resistance *in vivo* (Bailey *et al.*, 2012). Moreover, the importance of IFITM3 was highlighted by reports showing that an *IFITM3* allele (rs12252-C) is associated with enhanced disease severity caused by pandemic influenza virus H1N1/09 (Everitt *et al.*, 2012; Zhang *et al.*, 2013b) and highly pathogenic influenza virus H7N9 (Wang *et al.*, 2014) and is more frequent in Chinese than in Caucasian populations.

IFITM proteins comprise a relatively long hydrophilic N-terminal region, two hydrophobic intramembrane domains (IM1 and IM2) separated by a conserved intracellular loop (CIL), and a comparatively short hydrophilic C-terminal region. IM1 and CIL together constitute the CD225 domain, a functionally poorly defined domain shared by >300 members of the CD225/pfam04505 protein superfamily. Several alternative topologies have been proposed for the IFITMs (Chen *et al.*, 1984; Li *et al.*, 2013; Takahashi *et al.*, 1990; Yount *et al.*, 2012), but the most recent study suggests that murine Ifitm3 is a type II transmembrane protein, comprising an intracellular N terminus, an extracellular C terminus and a membrane-spanning ‘IM2’ domain (Bailey *et al.*, 2013). IFITM function is regulated by several post-translational modifications. *S*-Palmitoylation of IFITM3 on three membrane-proximal cysteine residues enhances membrane affinity and antiviral activity against influenza (Yount *et al.*, 2010, 2012). Conversely, lysine-linked ubiquitination decreases the co-localization of IFITM3 with endolysosomes and its antiviral potency (Yount *et al.*, 2012). The Y20 residue, which can be phosphorylated by the tyrosine kinase Fyn, is critical for targeting IFITM3 to endolysosomes and for restriction of endosome-dependent viruses (Jia *et al.*, 2012; John *et al.*, 2013).

Current evidence indicates that IFITMs block viral entry to the cytosol by preventing fusion between viral and host-cell membranes. IFITMs multimerize via their IM1 regions (John *et al.*, 2013) and increase membrane rigidity (Li *et al.*, 2013). This suggests a model in which IFITMs either physically resist the deformation of the membrane by the viral fusion machinery or hinder the lateral mobility of viral or cellular proteins within the membrane and thereby block successful pore formation (John *et al.*, 2013; Li *et al.*, 2013; Perreira *et al.*, 2013). Amini-Bavil-Olyaee *et al.* (2013) recently reported that IFITM3 interacts with a protein involved with cholesterol homeostasis, vesicle-membrane-protein-associated protein A (VAPA), and thereby causes cholesterol accumulation in multivesicular bodies and late endosomes, which inhibits the fusion between virion and endosomal membranes. However, a unifying mechanism to explain antiviral restriction by IFITM proteins remains elusive (Perreira *et al.*, 2013; Smith *et al.*, 2014). Indeed, recent reports showing that IFITM3 can be co-opted to promote cell entry of human coronavirus OC43 (Zhao *et al.*, 2014) and human papillomavirus (Warren *et al.*, 2014) suggest virus-specific IFITM interactions.

Although the *IFITM* gene family is evolutionarily conserved in vertebrates (Hickford *et al.*, 2012; Zhang *et al.*, 2012), it is unclear whether antiviral activity is also conserved among vertebrate IFITMs. Here, we focused on the bat and pig, as these hosts are particularly relevant to the ecology of several IFITM-sensitive zoonotic viruses.

## Results

### Sequence analysis of IFITM3 orthologues cloned from microbat and pig cells

In vertebrates, the *IFITM1*, -*2*, -*3* and -*5* genes cluster together in an *IFITM* locus flanked by the *B4GALNT4* and *ATHL1* genes. It was not possible to assign pig and bat IFITM3 orthologues based on conserved synteny due to the lack of a *B4GALNT4* orthologue in pigs, gaps in the genome assemblies and the low sequencing coverage of the bat genomes (2.6× for *Pteropus vampyrus* and 1.7× for *Myotis lucifugus* at the time of analysis). Therefore, to identify *IFITM* genes, rapid amplification of cDNA ends (RACE) was performed on a newborn pig tracheal cell line (NPTr) and primary lung fibroblasts from the greater mouse-eared bat, *Myotis myotis*, a European species of microbat (Vespertilionidae). *M. myotis* was selected as it is a known reservoir host for several highly pathogenic viruses (Amengual *et al.*, 2007; Drexler *et al.*, 2011, 2012) and genomes are available for other species from the same genus (Seim *et al.*, 2013; Zhang *et al.*, 2013a).

**Fig. 1.**
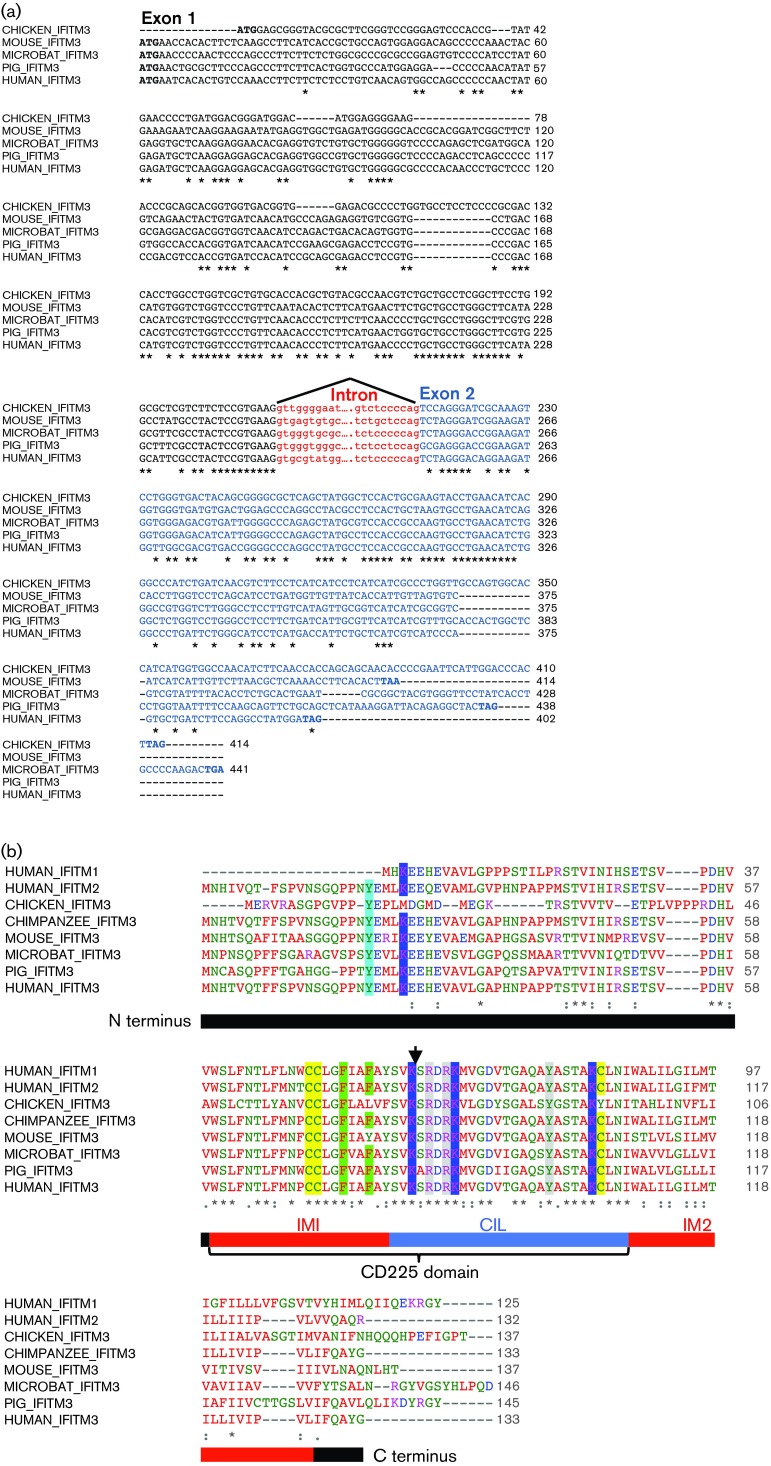
Multiple sequence alignments with microbat and pig IFITM3. (a) Pig and microbat IFITM3 nucleotide sequences were aligned with experimentally verified IFITM3 orthologues. The intron–exon boundary is shown (exon1: black; exon 2: blue) and intronic sequences flanking the splice sites are in red lower-case letters. An asterisk indicates identical nucleotides. (b) Pig and microbat IFITM3 amino acid sequences were aligned with other IFITM proteins. Text colour of amino acid residues denotes their physiochemical properties: blue: acidic, red: small and hydrophobic, magenta: basic, green: hydroxyl, sulfhydryl, amine and glycine. An asterisk indicates identical amino acid residues, ‘:’ indicates residues with strongly similar properties and ‘.’ indicates residues with weakly similar properties. The arrow indicates the exon–exon boundary. The protein domains of human IFITM3 are shown (according to John *et al.*, 2013): IM1, CIL and IM2. Highlighted residues are discussed within the text.

RACE using primers designed to the conserved central regions of compiled *IFITM*-like sequences yielded several *IFITM* gene variants, of which the designated IFITM3-like sequences were the most abundant. For the pig, the IFITM3-like sequence was the only IFITM variant identified that had an N-terminal extension typical of IFITM2/3 proteins (compare human IFITM1-3 in Fig. 1b). For the microbat, the sequence we assigned as IFITM3 was the most frequent of several long IFITM variants (68 % of sequenced clones) and the only one encoding a double phenylalanine motif (F8/F9) conserved in the human and pig IFITM3 orthologues but absent from human IFITM2 (Fig. 1b).

Full-length *IFITM3* cDNA sequences were obtained by reverse transcription-PCR, and introns were identified by PCR using genomic DNA, thereby confirming that these were not intronless expressed pseudogenes. The transcript structure for pig and microbat IFITM3 was the same as for other experimentally verified IFITM3 orthologues, comprising two exons, a single intron of similar size to other *IFITM3* genes and a conserved exon–exon junction site (Fig. 1a). blast searches revealed that the *IFITM3* sequence from NPTr cells was identical to the *Sus scrofa IFITM3* reference sequence (GenBank accession no. NM_001201382.1), and the closest match for the cloned microbat IFITM3 was a ‘predicted *IFITM3*-like’ gene from *M. lucifugus* (GenBank accession no. XP_006108229.1, 95 % amino acid identity).

Multiple sequence alignments showed that amino acid residues that are functionally important in murine and human IFITM3 were conserved in the cloned pig and microbat orthologues (Fig. 1b). These included: (i) three cysteine residues that are *S*-palmitoylated (Yount *et al.*, 2010); (ii) several lysine residues modified by ubiquitination (Yount *et al.*, 2012) (including K88, which can be monomethylated; Shan *et al.*, 2013); (iii) the Y20 residue critical for endosomal targeting (Jia *et al.*, 2012; John *et al.*, 2013); (iv) two phenylalanine residues (F75 and F78), which mediate oligomerization (John *et al.*, 2013); and (v) R85, R87 and Y99 shown to influence antiviral restriction (John *et al.*, 2013) (Fig. 1b, shaded residues). The microbat and pig IFITM3 proteins were most divergent from human IFITM3 at their N and C termini, and were most conserved in the central CD225 domain, a pattern shared with other orthologues (chicken and mouse Ifitm3) and paralogues (human IFITM2 and IFITM1) (Fig. 1b).

### Microbat and pig IFITM3 localize to transferrin- and CD63-positive endosomes

To analyse the IFITM3 proteins, A549 cells (which express low levels of endogenous IFITM3; Brass *et al.*, 2009) were stably transduced to express C-terminally haemagglutinin (HA)-tagged IFITM3 from pig, microbat or human. Prior work has shown that C-terminal epitope tags do not affect the function or expression levels of IFITM3, and indicate that tagged constructs adopt the same topology as the WT protein (Bailey *et al.*, 2013).

**Fig. 2.**
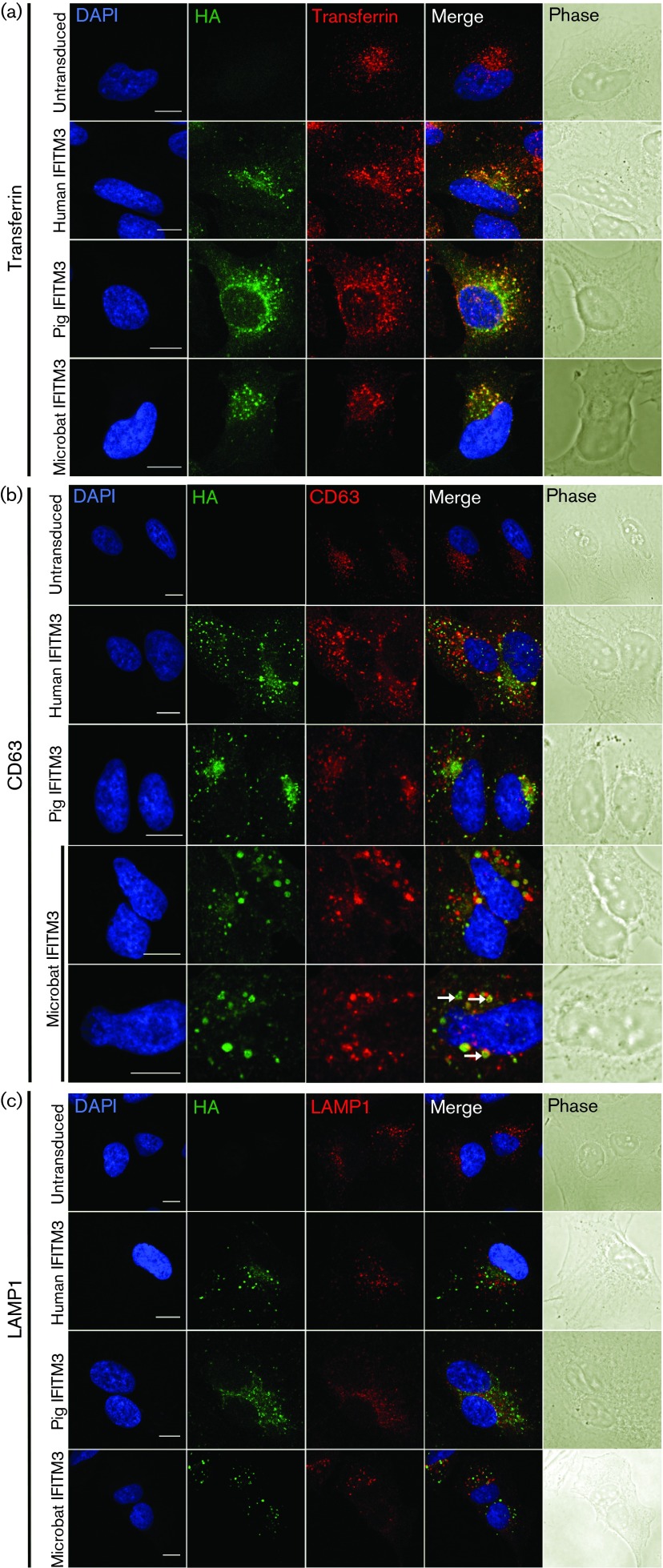
Subcellular localization of IFITM3 proteins. A549 cells stably expressing HA-tagged IFITM3 proteins from human, pig or microbat (or untransduced cells) were fixed, permeabilized and stained for HA, CD63 (b) or LAMP1 (c). In (a) cells were incubated with Alexa Fluor 546-conjugated transferrin before fixation and staining with FITC-conjugated anti-HA. Nuclei were stained with DAPI and the coverslips examined by confocal microscopy. Images show representative staining patterns, and merged images are also shown. The right-hand column shows phase-contrast images. Bar, 10 µm. Arrows in (b) identify ‘ring-like’ staining of microbat IFITM3–HA and CD63.

Similar to human IFITM3, both pig and microbat IFITM3 had a punctate intracellular distribution following cell fixation and permeabilization (Fig. 2) and co-localized with endocytosed transferrin (early endosomes) and with CD63, a marker for late endosomes/multivesicular bodies (MVBs), but not with the lysosomal marker LAMP1 (Fig. 2). There was an enlargement of CD63-positive structures in cells expressing microbat or human IFITM3 in comparison with the smaller CD63-positive vesicles seen in pig IFITM3-expressing or untransduced A549 cells (Fig. 2b). Enlargement of CD63-containing compartments was most marked in cells containing larger foci of IFITM3–HA staining. Microbat IFITM3–HA sometimes co-localized with CD63 in ‘hollow’ ring-like structures (arrows in Fig. 2b).

### Endocytic uptake of microbat and pig IFITM3 from the plasma membrane

**Fig. 3.**
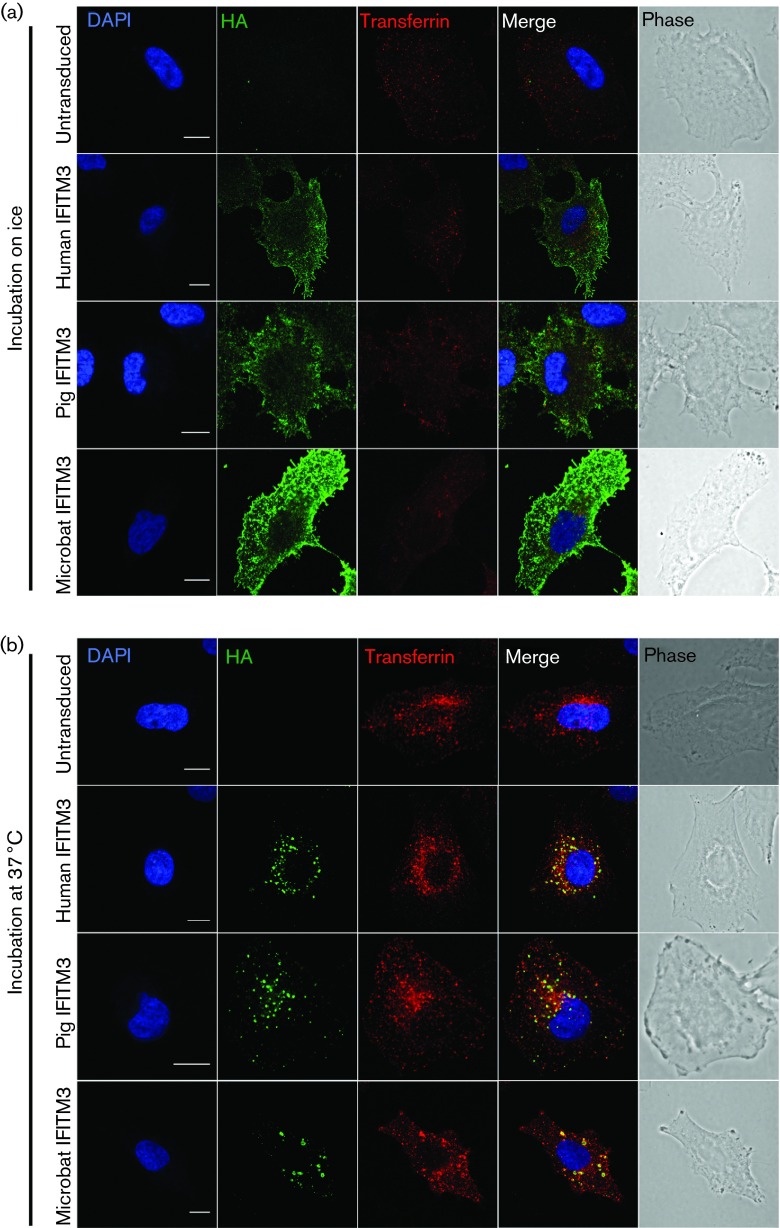
Endocytic uptake of IFITM3 proteins. Live A549 cells expressing HA-tagged IFITM3 from human, pig or microbat (or untransduced cells) were incubated either on ice (a) or at 37 °C (b) with FITC-conjugated anti-HA and Alexa Fluor 546-conjugated transferrin. After cell fixation, nuclei were stained with DAPI and the coverslips examined by confocal microscopy. The two right-hand columns showed merged images and phase-contrast images. Bar, 10 µm.

To investigate the trafficking of pig and microbat IFITM3 proteins, live cells were incubated with FITC-conjugated anti-HA antibody and Alexa Fluor 546-conjugated transferrin prior to fixation. When labelling was performed on ice, endocytosis was prevented, as indicated by the weak stippled transferrin signal, probably corresponding to clathrin-coated pits pre-internalization (Fig. 3a). Under these conditions, IFITM3 from the three species was detected on the plasma membrane, clearly highlighting filopodia and membrane ruffles, whilst no vesicular staining was observed. In contrast, when labelling was performed at 37 °C to allow endocytosis, anti-HA staining showed that pig, microbat and human IFITM3 formed discrete puncta, which, as observed previously, overlapped or were closely associated with transferrin-positive endosomes (Fig. 3b). For all IFITM3 proteins, internalization of the anti-HA antibody by live cells identified larger, brighter puncta that were more widely distributed within the cytosol compared with the HA staining seen previously following fixation and permeabilization (indicating that permeabilization may cause some extraction of the membrane-associated IFITM3). In conclusion, pig and microbat IFITM3 share the following with their human counterpart: (i) plasma membrane trafficking; (ii) extracellular exposure of their C termini (allowing detection of the C-terminal HA tag in intact cells); and (iii) endocytic uptake from the plasma membrane into the endosomal pathway.

### Microbat and pig IFITM3 restrict cell entry mediated by influenza HA

**Fig. 4.**
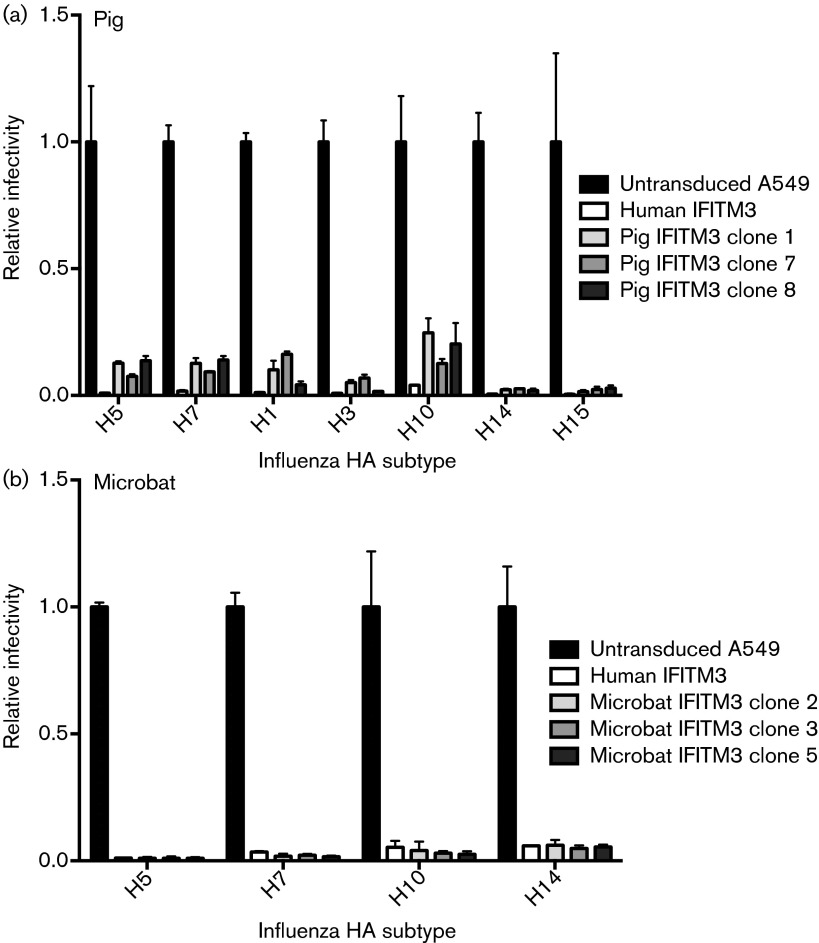
Microbat and pig IFITM3 inhibit influenza HA-mediated cell entry. A549 cells stably expressing IFITM3 from human, pig (a) or microbat (b) (or untransduced cells) were infected with pseudotyped viruses which express different influenza HA glycoproteins. At 48 h post-infection, luciferase reporter activity was measured and normalized to untransduced cells (relative infectivity of 1 corresponding to 3000–5000 relative light units according to pseudotype used). Results are shown as means±sd (*n* = 3), and data are representative of three independent experiments.

To determine whether pig and microbat IFITM3 restrict influenza A virus entry, lentiviruses expressing the HA proteins from diverse influenza subtypes were used to infect A549 cells that were untransduced or had been transduced to express either human IFITM3, pig IFITM3 (Fig. 4a) or microbat IFITM3 (Fig. 4b). Three independently cloned cell lines of the pig and microbat IFITM3 proteins markedly inhibited the infectivity of all influenza HA subtypes tested, including both group 1 and group 2 HAs and highly pathogenic avian H5 and H7 (Fig. 4). Pig or microbat IFITM3 did not restrict viruses pseudotyped with the entry proteins of the gammaretroviruses amphotrophic murine leukemia virus or Gibbon ape leukemia virus (Fig. S1, available in the online Supplementary Material), consistent with previous studies on human IFITM3 (Brass *et al.*, 2009; Huang *et al.*, 2011).

### Microbat and pig IFITM3 inhibit replication-competent influenza virus

**Fig. 5.**
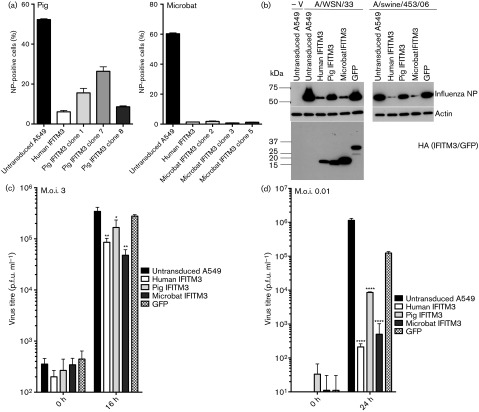
Microbat and pig IFITM3 inhibit influenza virus replication. A549 cells stably expressing human, pig or microbat IFITM3 were infected with influenza A virus. (a) At 7 h after infection with influenza A/WSN/33, NP expression was analysed by flow cytometry. (b) At 16 h after infection with influenza A/WSN/33 or A/swine/453/06 or mock infection (−V) cells were lysed and Western blotting was performed to detect influenza NP, actin and HA-tagged IFITM3 or green fluorescent protein (GFP). The molecular mass (kDa) of protein size markers is indicated. (c, d) Virus yields following infection with influenza A/WSN/33 at an m.o.i. of 3 (c) or an m.o.i. of 0.01 (d) were measured using plaque assays. Results are shown as means±sd (*n* = 3). In (b)–(d) pig IFITM3 clone 1 and microbat IFITM3 clone 2 were used. *P<0.05; **P<0.01; ****P<0.0001 relative to GFP-expressing cells (Student’s *t*-test).

To assess the effect of the IFITM3 orthologues on replication-competent influenza virus, untransduced A549 cells or cells expressing either human, pig or microbat IFITM3 were infected with influenza A/WSN/33 (H1N1) and nucleoprotein (NP) expression was quantified using flow cytometry. Expression of either pig or microbat IFITM3 led to a marked reduction in the proportion of NP-positive cells for all cell clones (Fig. 5a). There was a slight variation in the degree of restriction between the three cell lines expressing pig IFITM3 (Figs 5a and Fig. S2) despite comparable expression of IFITM3–HA seen by Western blotting (Fig. S2).

Stable expression of pig, microbat or human IFITM3 in A549 cells significantly reduced single-cycle growth of influenza A/WSN/33 (Fig. 5c), consistent with reduced NP expression measured in parallel (Fig. 5b). Inhibition of virus yields was more profound following a low m.o.i. infection (>1 log_10_ for pig IFITM3 and >2 log_10_ for both microbat and human IFITM3) relative to control green fluorescent protein (GFP)-expressing cells (Fig. 5d). A similar pattern of restriction was also observed when NP expression was analysed after infections with an avian-like swine influenza strain, A/swine/453/06 (H1N1) (Fig. 5b). Levels of the stably expressed proteins were examined by Western blotting against the C-terminal HA epitope tag, and showed that pig IFITM3, human IFITM3 and GFP expression were comparable, whilst microbat IFITM3 was more highly expressed (Figs 5b and S3).

### Microbat and pig IFITM3 block cell entry mediated by lyssavirus entry proteins

In light of data that suggest there may be virus-specific antiviral determinants of IFITM (John *et al.*, 2013; Zhao *et al.*, 2014), it was of interest to explore the antiviral spectrum of pig and microbat IFITM3, especially for viruses that naturally infect these species.

**Fig. 6.**
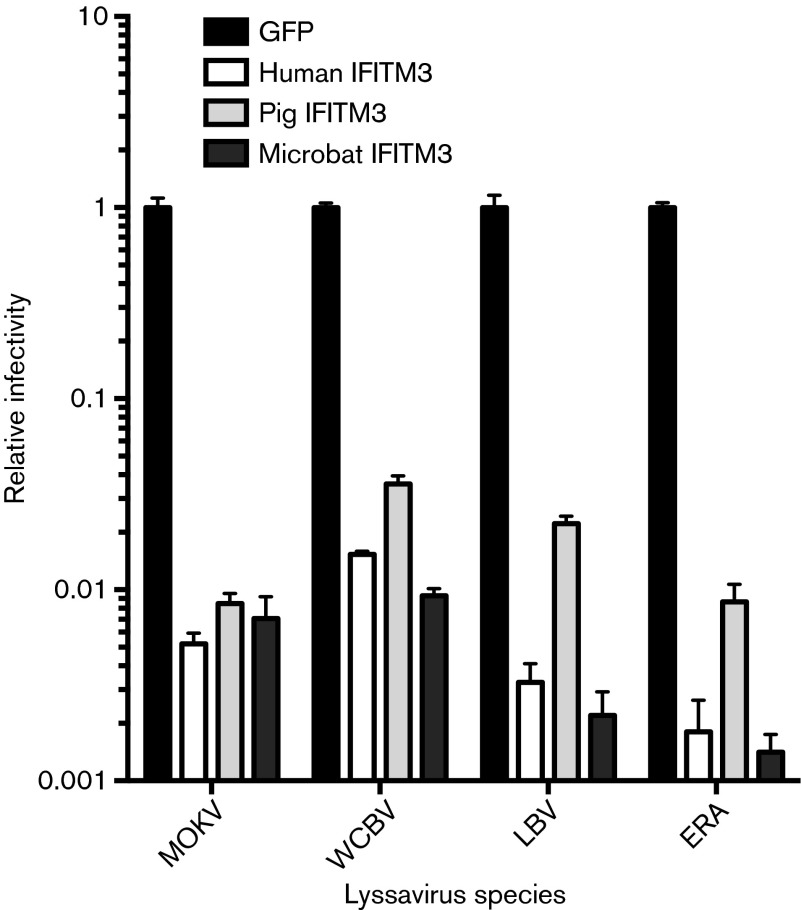
Microbat and pig IFITM3 inhibit cell entry mediated by lyssavirus G proteins. A549 cells stably expressing GFP, human IFITM3, pig IFITM3 (clone 1) or microbat IFITM3 (clone 2) were infected with pseudotyped viruses that expressed the envelope glycoprotein from Mokola virus (MOKV), West Caucasian bat virus (WCBV), Lagos bat virus (LBV) or rabies virus strain Evelyn Rokitniki Abelseth (ERA). At 48 h post-infection, luciferase reporter activity was measured and normalized to GFP-expressing cells (relative infectivity of 1 corresponding to 25 000–80 000 relative light units according to pseudotype used). Results are shown as means±sd (*n* = 3) and data are representative of three independent experiments.

A549 cells stably expressing either pig, microbat or human IFITM3 were infected with pseudotyped lentiviruses that expressed the envelope glycoproteins from isolates representing different lyssavirus phylogroups, namely rabies virus strain Evelyn Rokitniki Abelseth (phylogroup 1), Mokola virus (phylogroup 2), Lagos bat virus (phylogroup 2) and West Caucasian bat virus (phylogroup 3). Expression of IFITM3 from microbat, pig or human reduced infectivity mediated by all four lyssavirus entry proteins, in all cases by approximately 2 log_10_ relative to the control cells (Fig. 6). Thus, the pig and microbat IFITM3 orthologues inhibit cell entry mediated by multiple lyssavirus glycoproteins (G proteins).

### IFITM3 activity in pig and microbat cells

**Fig. 7.**
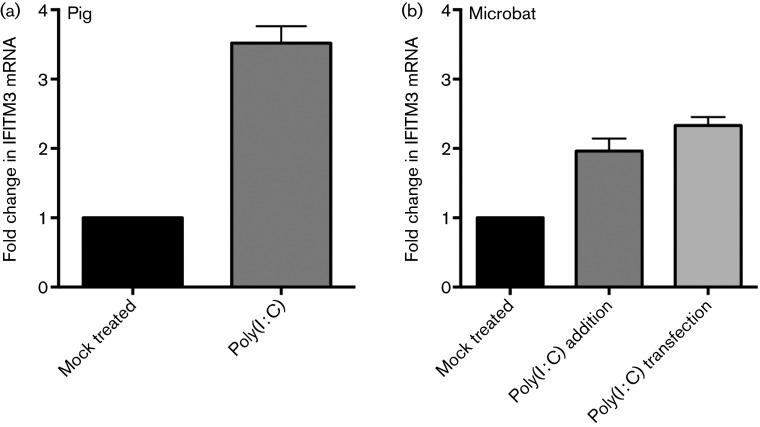
Microbat and pig IFITM3 are poly(I : C) responsive. Pig NPTr (a) and microbat (b) cells were either mock treated or stimulated by addition of poly(I : C) to the cell medium (50 µg ml^−1^) or transfection of poly(I : C) (33 µg ml^−1^) using Lipofectamine 2000 (microbat cells only). After 7 h, qRT-PCR was used to quantify IFITM3 and the reference gene, GAPDH. The fold change in IFITM3 mRNA is expressed relative to mock-treated cells. Results are shown as means±sd for biological triplicates assayed in duplicate.

Finally, we addressed the contribution of IFITM3 to antiviral responses in pig and microbat cells. Endogenous IFITM3 expression was measured using TaqMan quantitative reverse-transcription PCR (qRT-PCR) designed to specifically detect IFITM3 mRNA and not other IFITM paralogues identified in these cells by RACE. Baseline levels of IFITM3 mRNA were readily detected in both the porcine NPTr cells [threshold cycle (*C*_t_) value of IFITM3 = 22.7; *C*_t_ value of glyceraldehyde 3-phosphate dehydrogenase (GAPDH) = 21.33] and in microbat lung fibroblasts (*C*_t_ value of IFITM3  = 25.8; *C*_t_ value of GAPDH = 22.8). IFITM3 induction was assessed in response to the dsRNA analogue poly(I : C), a molecular pattern associated with viral infection, which is recognized by Toll-like receptor 3 and induces type I IFN in porcine (Provost *et al.*, 2012) and bat (Biesold *et al.*, 2011; Omatsu *et al.*, 2008; Zhou *et al.*, 2011) cells. Poly(I : C) addition led to a 3.5-fold increase in pig IFITM3 and a twofold increase in microbat IFITM3 mRNA levels (Fig. 7). The microbat cells were also stimulated via poly(I : C) transfection; as in pteropid bat lung cells (but not primary cells from other tissues) this enhanced IFN-β induction relative to extracellular delivery of poly(I : C) (Zhou *et al.*, 2011). However, transfection of poly(I : C) into microbat cells resulted in a similar degree of IFITM3 induction (2.3-fold) (Fig. 7).

**Fig. 8.**
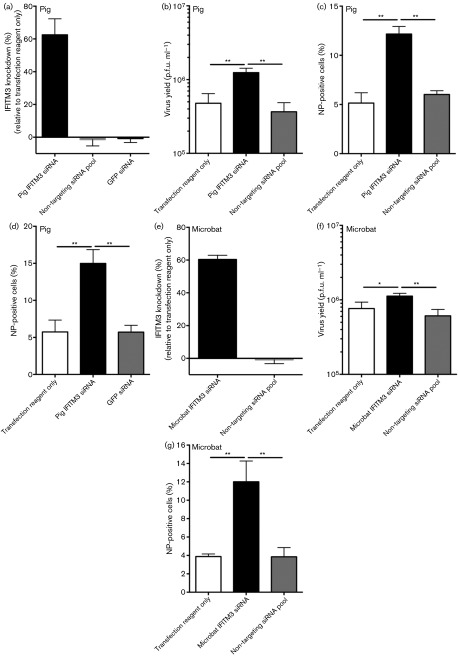
siRNA knockdown of IFITM3 in pig and microbat cells enhances influenza virus replication. Pig NPTr or microbat cells were transfected with siRNA targeting IFITM3 or control siRNA prior to quantification of IFITM3 by qRT-PCR (a, e) or infection with influenza A/WSN/33. Virus yields were measured by plaque assay (b, f) or NP expression was measured using flow cytometry (c, d, g). NPTr cells were either stimulated with poly(I : C) for 2 h before infection (m.o.i. 0.01) and analysed at 36 h post-infection (b, c) or cells were infected in the absence of poly(I : C). Results are shown as means±sd for biological triplicates assayed in duplicate and data are representative of two independent experiments. *P<0.05; **P<0.01 (Student’s *t*-test).

Next, the function of endogenous IFITM3 was assessed using small interfering RNA (siRNA) designed using the RACE sequence data to target IFITM3 and not other putative IFITM paralogues. siRNA targeting IFITM3 or control non-targeting siRNAs were transfected into cells with poly(I : C) induction of IFITM3. IFITM3 mRNA was quantified by qRT-PCR and the biological effect of the knockdown assessed by infection with influenza A/WSN/33 (Fig. 8). Transfection of siRNA against pig IFITM3 led to a 63 % knockdown in IFITM3 mRNA (fold change of 1.4 log_2_) (Fig. 8a), a 3.4-fold increase in virus yield (Fig. 8b) and a twofold increase in the proportion of influenza NP-positive cells (Fig. 8c) relative to control siRNA-transfected cells. In the absence of poly(I : C) stimulation, IFITM3 knockdown in NPTr cells increased influenza NP-positive cells more (3.3-fold) (Fig. 8d), suggesting that additional poly(I : C)-induced genes complement IFITM3-mediated influenza virus restriction. Microbat IFITM3 knockdown in the absence of poly(I : C) stimulation (Fig. 8e–g) reduced IFITM3 mRNA levels by 60 % (fold change of 1.3 log_2_) (Fig. 8e) and led to a twofold increase in infectious yields (Fig. 8f) and a threefold increase in NP expression (Fig. 8g), relative to control siRNA transfection. Thus, endogenous IFITM3 in pig tracheal NPTr cells and microbat lung cells restricts influenza virus. Lastly, following siRNA knockdown of baseline IFITM3 in a microbat cell line, overexpression of microbat IFITM3 significantly inhibited influenza NP expression (Fig. S4).

## Discussion

Species differences in restriction factors can determine differential viral susceptibility (Duggal & Emerman, 2012; Fadel & Poeschla, 2011; Kirmaier *et al.*, 2010; McNatt *et al.*, 2009). However, antiviral immunity in reservoir and spill-over hosts remains poorly understood, although important for understanding viral emergence (Bean *et al.*, 2013). Here, we showed that IFITM3 proteins with broad-spectrum antiviral function are conserved in swine and a member of the order Chiroptera, hosts of numerous zoonotic viruses.

The RACE reactions used here captured multiple possible expressed IFITM paralogues by using primers against the conserved CD225 domain. We assigned microbat IFITM3 using several criteria. First, it encoded a double phenylalanine motif (F8/F9) found only in other IFITM3 orthologues (whereas the other microbat IFITM variants resembled human IFITM2 in having a single phenylalanine at this position). Secondly, the microbat IFITM3 had an intracellular location and co-localized with endosomal markers. Conserved genome synteny was used previously to help assign *IFITM* genes (Smith *et al.*, 2013; Zhang *et al.*, 2012), but was of limited use in the case of the poorly assembled *IFITM* loci in the pig and microbat genomes. Moreover, the *IFITM* gene family is associated with numerous processed pseudogenes, gene duplications and copy-number variation (Siegrist *et al.*, 2011; Zhang *et al.*, 2012), which significantly complicate the assignment of gene orthology. Although a recent computational study identified eight pig *IFITM* family members with expressed sequence tag evidence (Miller *et al.*, 2014), it lacked the functional validation presented here.

Functionally important amino acid residues for human or mouse IFITM3 were conserved in pig and microbat IFITM3, indicating that these may be functionally important sites across the orthologues. Amino acid residues are less conserved within IM2 compared with IM1, although IM2 can function as a signal anchor for membrane localization (Bailey *et al.*, 2013) and is also sufficient to mediate the IFITM3–VAPA interaction (Amini-Bavil-Olyaee *et al.*, 2013).

We showed here that C-terminally HA-tagged IFITM3 (of human, pig and microbat) was clearly detectable at the plasma membrane after live cell staining. These data support recent evidence for a luminal (i.e. extracellular) exposure of the C terminus (Bailey *et al.*, 2013), and indicate that pig and microbat IFITM3 adopt a similar topology. Other studies have also reported that a proportion of human IFITM3 localizes to the plasma membrane (Amini-Bavil-Olyaee *et al.*, 2013; Bailey *et al.*, 2013; Brass *et al.*, 2009), and it is thought that the ^20^YEML^23^ motif acts as a lysosomal sorting signal for the internalization of IFITM3 into the endosomal pathway (Jia *et al.*, 2012; John *et al.*, 2013). Pig and microbat IFITM3 contain ^20^YEML^23^ and ^20^YEVL^23^, respectively (which conform to the consensus sequence for a tyrosine-based sorting signal YxxΦ, where Φ is a residue with a bulky hydrophobic side chain; Bonifacino & Traub, 2003), and likewise were observed to traffic into endosomes following their cell-surface staining. Pig and microbat IFITM3 co-localized with endocytosed transferrin (early endosomes) and CD63 (late endosomes/MVBs) as seen for human IFITM3 (Figs 2 and 3) (Amini-Bavil-Olyaee *et al.*, 2013; Feeley *et al.*, 2011; Huang *et al.*, 2011; Jia *et al.*, 2012; Lu *et al.*, 2011). Expression of both microbat and human IFITM3 caused expansion of CD63-positive endosomal compartments, consistent with the documented ability of IFITM3 to induce MVB formation (Amini-Bavil-Olyaee *et al.*, 2013). Furthermore, in some cells, microbat IFITM3 co-stained with CD63 in ring-like structures, a phenomenon reported for human IFITM3 and enhanced by overexpression of its interaction partner, VAPA (Amini-Bavil-Olyaee *et al.*, 2013). In our hands, neither human IFITM3 nor its pig and microbat orthologues co-localized with the lysosomal marker LAMP1, which is consistent with some (Yount *et al.*, 2010) but not other (Feeley *et al.*, 2011; Huang *et al.*, 2011) reports for IFITM3 localization. These discrepancies regarding localization may be due to the multiple post-translational modifications of IFITM3 (Yount *et al.*, 2012) and/or cell type-dependent differences in its topology (Bailey *et al.*, 2013).

We showed that both pig and microbat IFITM3 restricted cell entry mediated by multiple influenza A virus HA (a class I fusion protein) and lyssavirus G (a class III fusion protein) proteins. Restriction at the level of cell entry correlated with significant inhibition of influenza virus yields and NP expression. The microbat, pig and human IFITM3-expressing A549 cells varied in their IFITM3 expression levels, which may underlie variation seen in the degree of restriction. However, anti-influenza restriction by pig IFITM3 was in general lower than that seen for human IFITM3, despite comparable expression levels. We found that pig and human IFITM3 could restrict avian, swine and human influenza A subtypes. Similarly, chicken IFITM3 inhibited viral pseudotypes bearing HAs from both avian and human strains (Smith *et al.*, 2013). siRNA knockdown of endogenous pig and microbat IFITM3 enhanced influenza replication by a similar degree to that seen following knockdown of human IFITM3 (Huang *et al.*, 2011) or chicken IFITM3 (Smith *et al.*, 2013). IFITM3 was expressed constitutively in pig and bat cells (as reported for other IFITM3 orthologues; Bailey *et al.*, 2012; Everitt *et al.*, 2012, 2013; Friedman *et al.*, 1984; Smith *et al.*, 2013). Baseline levels of IFITM3 in the pig and bat cells were sufficient to limit virus replication and, following siRNA targeting of baseline IFITM3, microbat IFITM3 was also capable of restricting influenza virus when overexpressed in microbat cells. As pig IFITM3 was moderately induced by poly(I : C) and upregulated upon viral challenge *in vivo* (Andersson *et al.*, 2011; Miller *et al.*, 2014), pig IFITM3 is likely to be relevant to host antiviral responses.

Here, we showed that influenza A viruses and lyssaviruses, virus families that share an ancient co-evolutionary history with bats (Badrane & Tordo, 2001; Tong *et al.*, 2013), are restricted by microbat IFITM3. Bats harbour many diverse virus types and are important reservoirs of zoonotic infections (Drexler *et al.*, 2014; Quan *et al.*, 2013; Smith & Wang, 2013; Tong *et al.*, 2013). However, the basis for the intimate association between bats and viruses remains enigmatic, particularly the relative importance of immunological compared with ecological or life history factors (Kupferschmidt, 2013). Although transcriptional induction of chiropteran IFN-stimulated gene orthologues has been reported (Papenfuss *et al.*, 2012; Zhou *et al.*, 2013), there is a striking lack of functional data for any of these genes. We demonstrated that bats do encode functional IFITM3 and therefore are likely to be competent in this aspect of intrinsic antiviral restriction.

## Methods

### 

#### Cells.

A549 cells were maintained in F12 medium, and 293T, NPTr (Ferrari *et al.*, 2003) and FLN-R cells (Friedrich-Loeffler-Institut) were grown in Dulbecco's Modified Eagle's Medium (DMEM) with 10 % FCS. Primary lung cells from the microbat (*M. myotis*) were grown in DMEM containing 10 % FCS, penicillin (100 units ml^−1^) and streptomycin (100 µg ml^−1^) and 20 % amnioMAX (Gibco).

#### RACE and IFITM3 cloning.

NPTr and microbat cells were transfected with poly(I : C) (Invivogen) at 33 µg ml^−1^ using Lipofectamine 2000, and 4 h later, total RNA and genomic DNA were isolated (Qiagen AllPrep kit). 5′ and 3′ IFITM cDNA fragments were generated using a SMARTer RACE cDNA amplification kit (Clontech) and the following primers: 5Bat: 5′-GCCCAGAGCTATGCGTCCACCGCCAAGTGCC-3′, and 3Bat: 5′-CGTCTGGTCCCTGTTCAACACCCTCTTC-3′; and 5Pig: 5′-GATGTTCAGGCACTTGGCGGTGGAGGCATAGCTC-3′, and 3Pig: 5′-TGAACTGGTGCTGCCTGGGCTTCGTGG-3′. PCR products were TA cloned and multiple clones were sequenced using Sanger sequencing. Non-coding sequences identified by RACE were used to design primers for PCR amplification of full-length IFITM3 (BatIFITM3ncrF: 5′-GCATCCACACGCCATCTGCTC-3′, and BatIFITM3ncrR: 5′-GAACGCCATTGTGCACATGTGC-3′; and PigIFITM3ncrF: 5′-ACAGCTTCTCCTGGGCACCATG-3′, PigIFITM3ncrR: 5′-GTATGTGCTGCTGTGAAAGGAG-3′). Pig IFITM3 and microbat IFITM3 were synthesized as codon-optimized genes for expression in human cells (GeneArt) and cloned into the *Bam*HI and *Not*I sites of the lentivirus vector pSIN-BNHA (derived from pHRSIN-CSGW; Demaison *et al.*, 2002).

#### Creation of IFITM3-expressing cell lines.

Lentiviruses expressing IFITM3 (or GFP as a control) were produced by co-transfecting 293T cells using Fugene HD with the packaging plasmid p8.91 (Zufferey *et al.*, 1997) (1 µg), the VSV-G-expressing plasmid pMDG (1 µg) and the lentivirus vector pSIN-BNHA (1.5 µg) containing either pig IFITM3, microbat IFITM3 or GFP. Supernatants were harvested at 48 and 72 h, filtered (0.45 µm) and used to transduce A549 cells. Transduction efficiency was checked after 48 h by flow cytometric detection of HA. Single-cell clones were generated using limiting dilution and analysed for HA expression. Those with the most similar expression levels were selected for experiments. Human IFITM3-expressing A549 cells were generated using the same method (Smith *et al.*, 2013).

#### Pseudotyped lentivirus cell entry assay.

Lentiviral pseudotypes were produced as described previously (Ferrara *et al.*, 2012; Wright *et al.*, 2008). For the entry assay, A549 cells were seeded in white 96-well plates (10^4^ cells per well) 1 day prior to infection with luciferase-expressing pseudotypes expressing influenza HA (GenBank accession nos: H1, AAD17229.1; H5, ABP51969.1; H10, ABI84534.1; H14, BAF43460.1; H7, CAD37074.1; H3, AAA43099.1; H15, AAA96134.1), the G proteins from Mokola virus (MOKV.98/071 RA361; GQ500108), Lagos bat virus (LBV.NIG56-RV1; HM623779), West Caucasian bat virus (AAR03484) or rabies virus Evelyn Rokitniki Abelseth strain (ABN11294) or amphotrophic murine leukemia virus (MLV-A) or gibbon ape leukemia virus (GALV) envelope proteins. The H1 and H3 HAs were human in origin, and the other HA subtypes were avian in origin. Cells were infected in triplicate, and 48 h later, luciferase activity was measured using a Bright-Glo Luciferase Assay System (Promega).

#### siRNA transfection and qRT-PCR.

siRNAs against pig IFITM3 (5′-GCTCATAAAGGATTACAGA-3′) or microbat IFITM3 (5′-CGAGGACGACGGTGGTCAA-3′) (ON-TARGETplus; Dharmacon) were designed using knowledge of other IFITM paralogues and the Dharmacon siDesign Centre. The ON-TARGETplus non-targeting siRNA pool and an siRNA targeting GFP were used as controls. siRNA was transfected into cells (20 pmol per well of a 12-well plate) using Lipofectamine RNAiMAX. After 48 h, RNA was extracted (RNeasy kit; Qiagen) and a Quantitect multiplex RT-PCR kit (Qiagen) was used to measure *IFITM3* and *GAPDH* simultaneously using TaqMan gene expression assays (Applied Biosystems; primer sequences available on request). MxPro software was used for comparative quantification of *IFITM3* relative to the *GAPDH* reference gene.

#### Flow cytometry.

Cells were infected in triplicate with influenza A/WSN/33 in medium containing 2 % FCS. Cells were trypsinized, fixed using BD Fixation/Permeabilization solution for 20 min and washed twice in BD Perm/Wash buffer before incubation with FITC-conjugated anti-influenza NP IgG (Abcam) for 40 min at 4 °C. After staining, the cells were washed, resuspended in PBS and analysed using a Becton Dickinson FACSCalibur and Cell Quest Pro. For each sample, 10 000 cells (gated by forward and side scatter) were analysed for FITC fluorescence.

#### Western blotting.

After cell lysis using RIPA buffer, proteins were separated by SDS-PAGE (4–12 % TGX gel; Bio-Rad) and transferred to nitrocellulose membrane. After blocking (5 % Marvel, 0.1 % Tween 20 in PBS), the membranes were incubated for 1 h at room temperature with antibodies against influenza NP, β-actin or the HA tag (clone HA.C5; all from Abcam). HRP-conjugated secondary antibodies were used followed by enhanced chemiluminescent detection.

#### Virus yield assay.

Cells were infected in triplicate with influenza A/WSN/33 in medium containing 2 % FCS. After 1 h infection at 37 °C, the cells were washed three times in PBS. At the indicated times post-infection, supernatants were harvested and the virus yields titrated on MDCK cells by plaque assay (Matrosovich *et al.*, 2006).

#### Immunofluorescence.

Cells were fixed with 4 % paraformaldehyde (20 min at room temperature). For transferrin co-localization, cells were pre-incubated with Alexa Fluor 546-conjugated transferrin (5 µg ml^−1;^ Molecular Probes) for 10 min before fixation. Cells were permeabilized using 0.2 % Triton X-100, blocked using antibody buffer (0.1 % Tween 20 and 10 % goat serum in PBS) and stained with FITC-conjugated anti-HA (Bethyl Laboratories), anti-LAMP1 (clone H4A3; Abcam) or anti-CD63 (Santa Cruz Biotechnology) for 1 h at room temperature. Dylight 594-conjugated goat anti-mouse IgG was used to detect the anti-CD63 and anti-LAMP1 antibodies. Live cell staining was performed at 37 °C or on ice, with solutions equilibrated accordingly. Cells were washed twice in serum-free medium before incubation for 30 min with FITC-conjugated anti-HA and Alexa Fluor 546-conjugated transferrin (in serum-free medium). Cells were then washed quickly in PBS and fixed immediately using 4 % paraformaldehyde (20 min). Coverslips were mounted using ProLong Gold reagent with DAPI and examined with a Zeiss LSM 780 confocal microscope.

#### Sequence analysis.

Multiple sequence alignments were performed using Clustal Omega (version 1.2.0), and Seaview (Gouy *et al.*, 2010) was used for manual editing.
